# Assessing the effects of limited vaccine supply on risk-based vaccination strategies in regions of endemic foot-and-mouth disease

**DOI:** 10.1186/s13567-026-01805-y

**Published:** 2026-07-07

**Authors:** Glen Guyver-Fletcher, Mike Tildesley

**Affiliations:** Zeeman Institute for Systems Biology and Infectious Disease Epidemiology Research (SBIDER), School of Life Sciences and Mathematics Institute, University of Warwick, Coventry, UK

**Keywords:** FMD, epidemiology, vaccines, control, optimisation, simulation, policy

## Abstract

Foot-and-mouth disease (FMD) is an important livestock disease that is endemic in many regions of the world, many of which struggle to supply enough vaccine doses to cover the entire population of animals at risk. We use a previously fitted stochastic metapopulation model of FMD spread, utilising high-quality data from Erzurum in Turkey, to simulate four plausible vaccine allocation strategies in the context of limited vaccine supply and endemic disease. We find that random allocation outperforms other strategies when the objective is to minimise average prevalence, and random allocation also achieves local eradication at lower doses per capita. However, all strategies perform similarly when minimising annual incidence, with the ‘optimal’ allocation strategy varying by doses available per capita. These results suggest that policymakers in regions of endemic FMD should focus more on sourcing high-quality vaccines and achieving high coverage, with risk-based vaccination being a secondary concern.

**Supplementary Information:**

The online version contains supplementary material available at 10.1186/s13567-026-01805-y.

## Introduction

Foot-and-mouth disease (FMD) is one of the most infectious livestock diseases known, infecting a large variety of cloven-hoofed animal species, including major livestock animals such as cattle, buffalo, sheep, goats, and pigs [1]. The aetiological agent is a virus, foot-and-mouth disease virus (FMDV), of the *Apthovirus* genus and *Picornaviridae* family. Symptoms vary by species, but common symptoms include lesions around the feet and mouth, fever, and lameness. In cattle, reductions in milk production are also common [2]. The disease is endemic in most countries in Africa and Asia, and it has been estimated that visible production losses due to FMD and vaccination in endemic areas alone amount to between $6.5 and $21 billion USD annually [3]. More recent attempts estimate USD$3.2 billion in annual losses in India alone, assuming severe incidence scenarios [4]. However, estimates in Ethiopia only found mean annual losses of 0.9 million USD, suggesting that subsistence-oriented economies may be less exposed to FMD-related damage [5].

Efforts to limit or eradicate the spread of FMD are common; historically, control efforts have been successful in Europe and South America; however, such efforts relied on mass vaccination campaigns [6, 7]. For many countries where the disease is currently endemic, procurement of efficacious vaccines matched to locally circulating variants remains problematic. Even if such vaccines are available, their expense may be prohibitive, leading to smaller numbers of doses being procured than are necessary to vaccinate all animals at risk [8].

Given that vaccine supply is limited in many settings, it is important to ensure that doses are allocated in the most effective way. This scenario – where the supply of efficacious vaccine doses is insufficient to meet demand – may have knock-on effects on the allocation of those doses; the optimal allocation when many doses are available may be different from the optimal allocation when few doses are available.

Epidemiological modelling allows for the exploration of optimal vaccine dose allocation strategies. Previous work has generally focused on optimising vaccination policies against epidemics of FMD [9–11] although there has been limited work on simulating optimal control policies in endemic areas [12, 13].

We therefore aim to use a previously developed and validated simulation model of FMD spread in endemic areas to investigate how the ‘optimal’ allocation of vaccine doses changes as the availability of doses changes. We focus on Turkey owing to its combination of endemic FMD and high-quality data necessary for detailed simulation. Specifically, we wish to know whether: (1) The ‘optimal’ strategy changes at different dose availabilities; and (2) which strategies are most optimal, for each criterion investigated.

## Materials and methods

### Data

Data are available from Turkey 2001–2012, comprising: (i) village point locations and cattle headcounts; (ii) village outbreak records, 2001–2012; (iii) village-to-village cattle movement records, 2007–2012. Owing to the detailed data and model and to keep simulation times reasonable, we limit our analysis to a single province, Erzurum. To provide a sense of the skewed distributions and network heterogeneity in the Erzurum movement records, we provide summary statistics in Table 1.

**Table 1:** **Descriptive statistics of Erzurum cattle movement network**.

Statistic	Minimum	25^th^ percentile	Mean	75^th^ percentile	Max
Headcount moved	1.0	1.0	2.29	2.00	924
Out-degree	1	79	227	300	2311
In-degree	1	48	227	255	8106
Movements-per-day	0	30	117	161	1237

All statistics show very heterogeneous distributions and long tails. Out-degree refers to the number of outgoing movements per village, in-degree the number of incoming cattle movements.

### Model

The model we use has been developed in the context of previous work on FMD in Turkey; for a more in-depth account of the model and parameters used, please see our previously published article [13]. We use the joint posterior parameter distributions from that work to simulate our model here.

**Figure 1:**
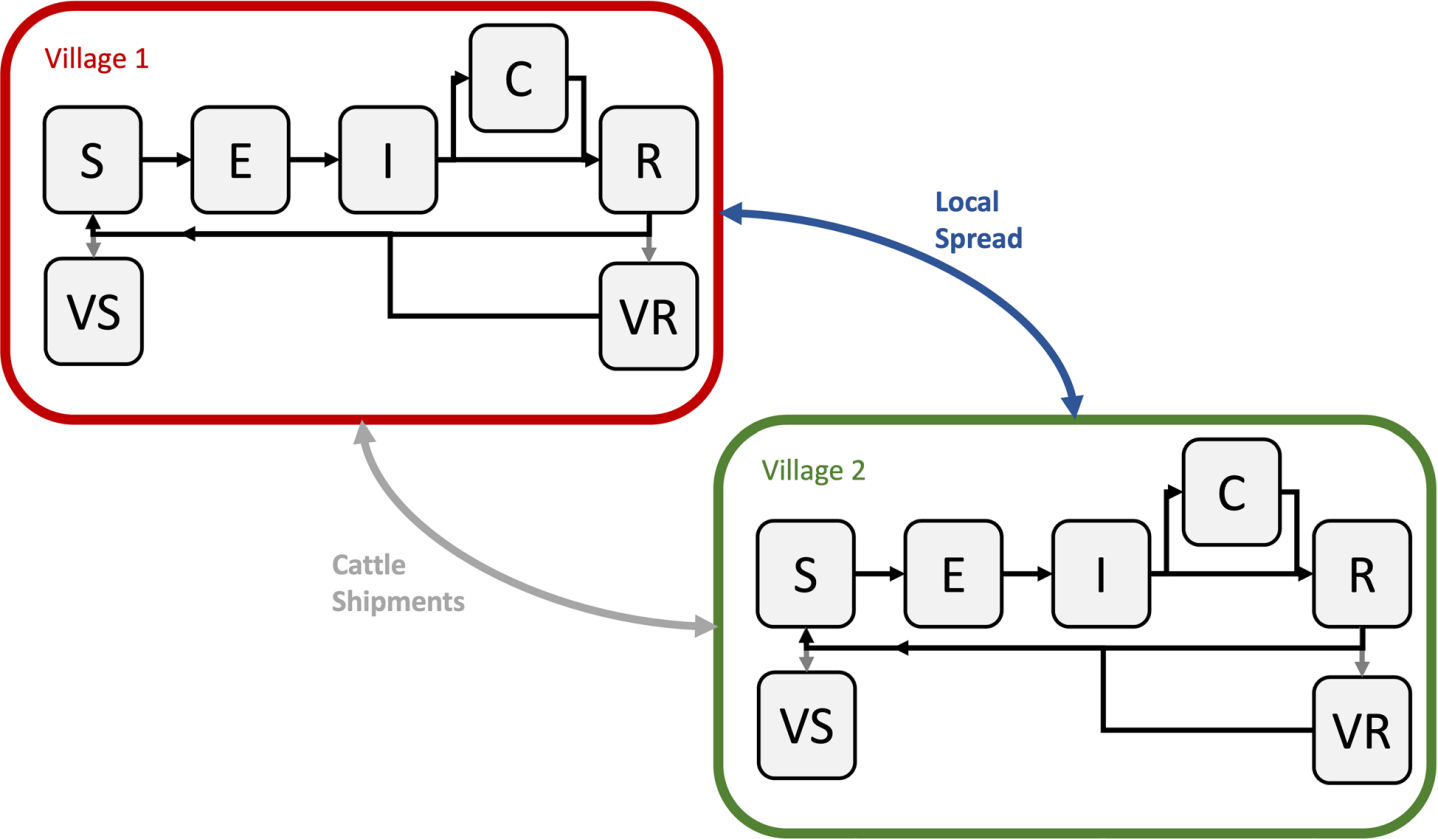
**Schematic representation of intra- and inter-village transmission.** Cattle proceed through disease states within a village at certain rates, defined by ODEs. Transmission between villages is simulated through two different routes: local transmission in the form of a distance-dependent kernel and explicit simulation of cattle shipments.

In summary, the model is a stochastic metapopulation model which simulates intra- and inter-village FMDV transmission. A schematic representation of the main model aspects is illustrated in Figure 1. Cattle within a village can be in one of eight disease-relevant states: maternally immune (M), susceptible (S), exposed (E), infectious (I), recovered (R), carrier (C), vaccinated-susceptible (V_S_) and vaccinated-recovered (V_R_). Rates of progression between these states is determined by Ordinary Differential Equations (ODEs) and implemented stochastically using the tau-leap algorithm. It is assumed that carrier animals do not transmit due to the lack of evidence for this in the field [14].

Disease transmission between villages is simulated through two routes: (1) direct shipments of cattle, with probability of transmission equalling the probability one of the animals moved is infected; (2) local transmission, aggregating aerosol transmission and other unseen routes of transmission (e.g. delivery trucks conveying fomites between village). The local transmission is estimated through a distance-dependent kernel function that scales the probability of transmission with the number of infectious animals in the source village and the number of susceptible animals in the "target" village.

### Vaccine allocation strategies

We define *δ*_v_ as doses per capita – the ratio of available vaccine doses to the time-varying total cattle population *N*_*t*_ at the commencement of each vaccination campaign. For example, a value of *δ*_v_ = 0.5 indicates a ratio of 1 available vaccine dose per two animals. For simplicity, we consider only application of a single dose at a time.

We simulate mass vaccination campaigns every 182 days (6 months). No other control policies are simulated. At campaign commencement, the number of available doses is calculated as ${\delta}_{v}\times {N}_{t}$, where *N*_*t*_ is the total population of cattle in existence at time *t*.

When simulating a vaccination campaign, the relevant epidemiological unit is the village to retain operational realism. We assume all animals in a village are targeted for vaccination, with the proportion achieving protection drawn from a normal distribution with mean equal to vaccine efficacy and standard deviation set to 5%. We order each village by the relevant criteria defined by the allocation strategy outlined in Table 2 and then vaccinate in order, subtracting the number of used doses, until there are either no doses left or no villages remaining to vaccinate. "Random" allocation, the comparison allocation strategy, consists of vaccinating villages with no targeting by size, connectedness or local density. We do not consider randomisation at the individual-animal level, as partial vaccination of herds is not operationally realistic for mass FMD vaccination campaigns. For model simplicity, we also do not consider logistical delays in implementing vaccination, such as travel or time taken to vaccinate herds.

**Table 2:** **Vaccine allocation strategies investigated**

Allocation strategy	Ordering criteria
Random	None – random
Population	Villages with the greatest cattle headcount are vaccinated first
Degree	Villages with the greatest number of recorded inward or outward cattle movements are vaccinated first
Density	Villages with the greatest number of other villages within 5 km are vaccinated first

### Simulation of strategies

To account for parameter uncertainty, we sample 100 particles from the posterior distribution of our model fit to the observed history of outbreaks in Erzurum province [13]. We simulate a no-control scenario with each particle, and 10 infections seeded at the start of the timeline, for 5 years to achieve a realistic endemic state.

For each endemic scenario, we then simulate a 5-year period for each *δ*_v_ in the sequence {0.0, 0.01, 0.02, …, 0.99}. Each combination of particle and *δ*_v_ is simulated 10 times. Each strategy is therefore simulated 1000 times per value of *δ*_v_ (100 particles, simulated 10 times per value of *δ*_v_).

We simulate vaccine efficacy (VE) value drawn from a normal distribution centred on 71% [15], or centred on 90%; in both cases, the standard deviation used was 5%.

For each combination of *δ*_v_ and allocation strategy we calculate the average annual village-level incidence and average prevalence over the simulated period, as well as the animal-level prevalence. We also calculate the probability of eradication as the proportion of simulations where eradication is achieved, and the time to eradication when or if eradication occurs is the first day when eradication is achieved, averaged over all simulations with the same *δ*_v_ and strategy.

### Sensitivity analysis

To investigate the sensitivity of our outputs to certain control parameters, we conducted a global sensitivity analysis using Latin Hypercube Sampling and Partial Rank Correlation Coefficients (LHS-PRCC). For each of the four strategies, we took 30 random samples from our posterior distribution and 80 samples from a Latin Hypercube generated with the joint control parameter distribution outlined in Table 3. We then simulated with a dose ratio *δ*_v_ of either 0.3 (low availability), 0.6 (medium availability) or 0.9 (high availability) to investigate how sensitivity to control parameters might differ in different dose availability regimes.

**Table 3:** **Marginal distributions of control parameters explored for the sensitivity analysis**

Control parameter	Description	Marginal distribution
Vaccine time to effect	The delay after vaccination before an animal is protected	Uniform (3–14) days
Vaccine efficacy	What proportion of animals generate protective immunity after vaccination	Uniform (1–100) percent
Vaccine duration	The average duration of protective immunity generated by the vaccine	Uniform (150–210) days
Mass vaccination coverage	The proportion of villages that are covered by the mass vaccination campaign	Uniform (0–100) percent
Mass vaccination interval	The number of days between mass vaccination campaigns	Uniform (120–240) days
Within-village coverage	The proportion of a village truly vaccinated, reflected in doses	Uniform (50–100) percent
Network scaling	How many animals are moved, as a scalar multiple of the recorded movement	Uniform (0.5–2)

Once we had simulated each particle, we calculated our outputs of interest and calculated the Partial Rank Correlation coefficients (PRCC) using the *epiR* package version 2.0.80 [16] in R 4.4.3 (R Core Team, 2025).

## Results

**Figure 2:**
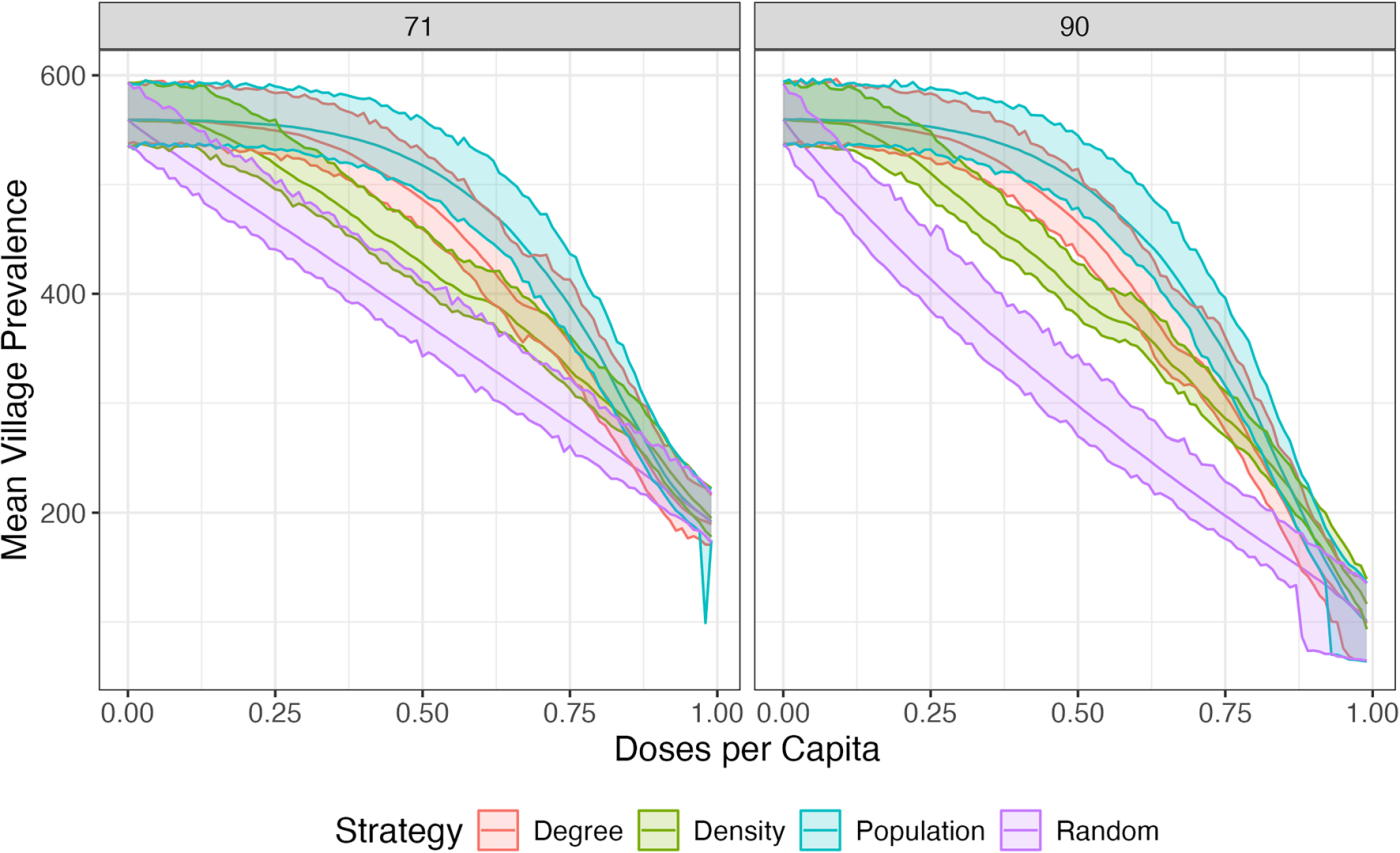
**Average village-level prevalence by doses, strategy and vaccine efficacy (panels).** Minimum and maximum simulated values are indicated by the shaded area around each line. Prevalence with the random allocation strategy declines linearly as more doses become available, at both values of VE. Allocation by any other strategy exhibits a non-linear curve in predicted prevalence, initially flatter below δv = 0.5, before declining much faster.

**Figure 3:**
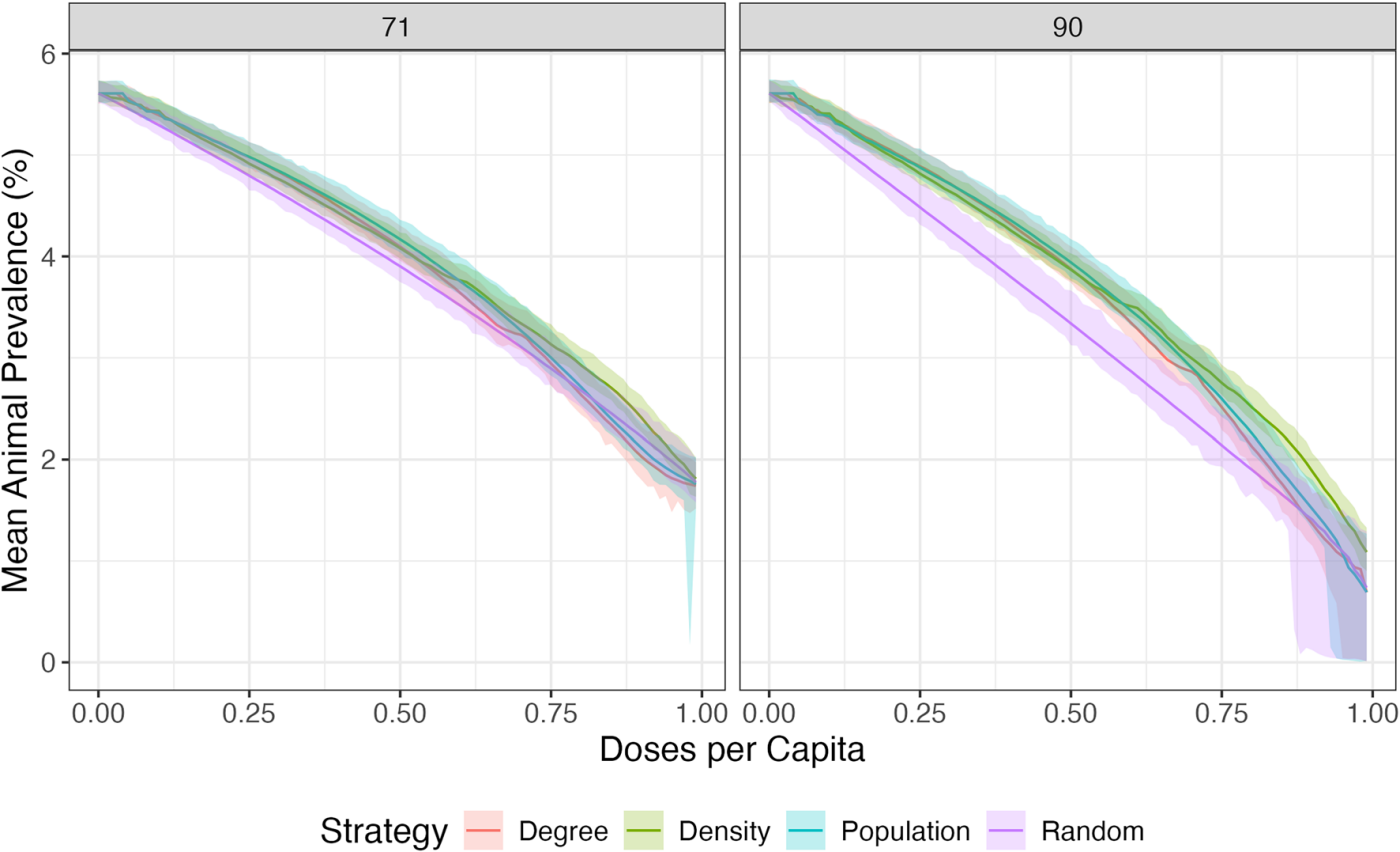
**Average animal-level prevalence by doses, strategy and vaccine efficacy (panels).** Minimum and maximums of the mean across simulation are indicated by the shaded areas. Prevalence with the random allocation strategy declines\*n* linearly as more doses become available, at both values of VE. Allocation by any other strategy exhibits a non-linear curve in predicted prevalence.

Prior to vaccination, prevalence with no controls ranges from 614 to 701 infected villages dependent upon parameter values; there is significantly lower prevalence in the north-west compared with all other areas, at 34% prevalence versus 63–65% prevalence elsewhere. Mean simulated prevalence declines with increasing availability of vaccine doses, as expected (Figure 2). At the majority of simulated *δ*_v_ values, the random allocation strategy leads to a lower mean simulated prevalence than using any other strategy, with all strategies converging as availability approaches 100%. Increased vaccine efficacy leads to a faster and larger decline. There is a small difference in observed trends between village- and animal-level prevalence (Figure 3) – the magnitude of performance differences between strategies (in terms of animal prevalence) is much smaller than their performance differences in terms of village prevalence; however, the overall ranking of strategies remains similar across the range of simulated doses per capita.

**Figure 4:**
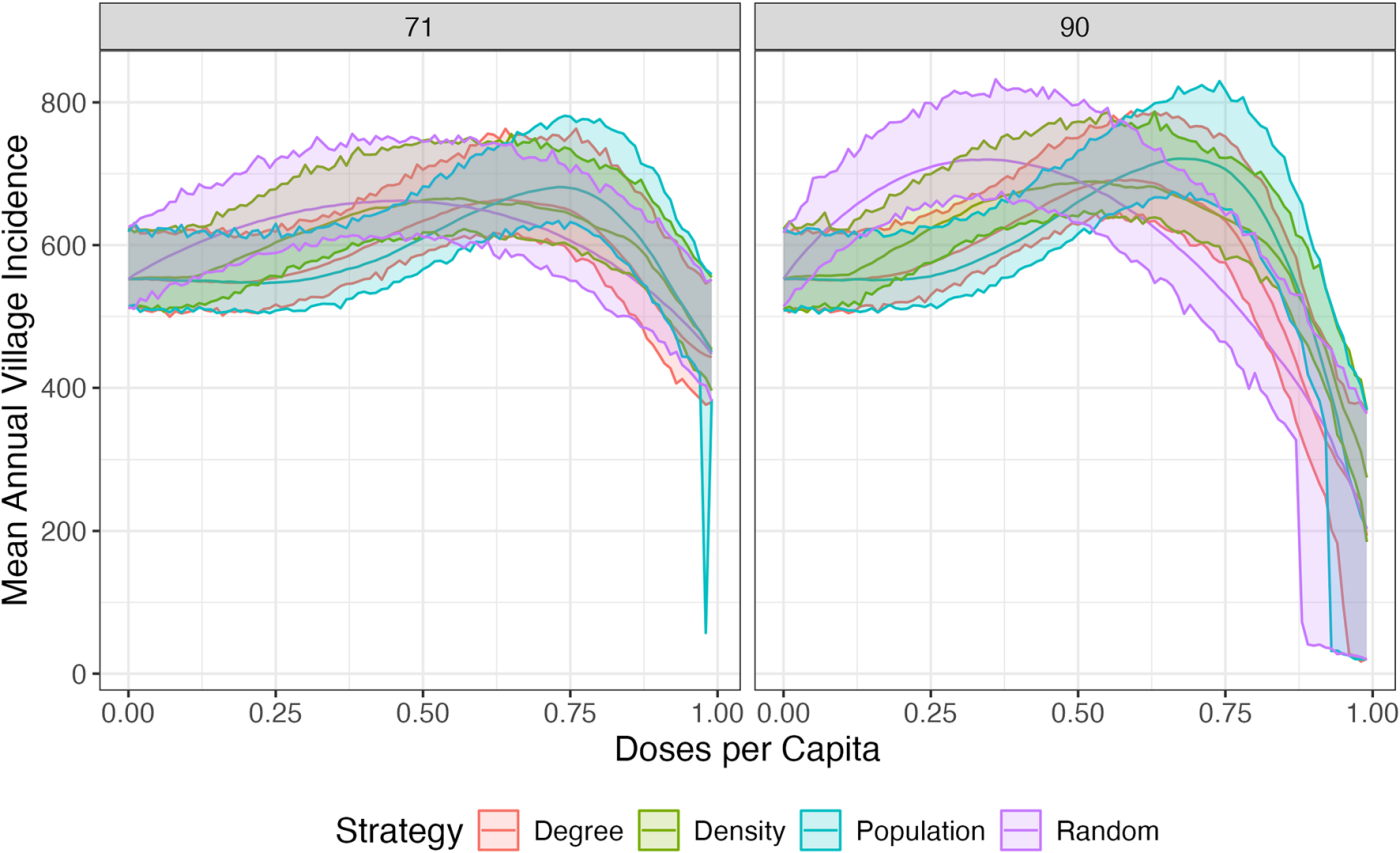
**Mean annual incidence by dose availability ratio, allocation strategy and vaccine efficacy (panels).** Minimum and maximum predicted values are indicated by the shaded areas. Mean annual incidence increases with greater dose availability for all allocation strategies, before declining, with the inflection point varying by allocation strategy and VE.

Figure 4 demonstrates a different ranking, with the population-based allocation strategy outperforming all others when *δ*_v_ < 0.6, and random allocation performing best above *δ*_v_ = 0.6. Mean annual incidence with all strategies is relatively flat or increasing until dose availability exceeds 0.75. Ranking of strategies is very similar between different vaccine efficacies. As dose availability approaches 1, mean annual incidence for population, degree and random allocation strategies declines towards (without reaching) 0 when VE = 90; however, when VE = 71 the decline is much smaller.

No strategy, with vaccine efficacy averaged at 71%, exhibited regular eradication of disease circulation; hence, probability of eradication is 0 and TTE is not applicable. The exception is a single simulation with the population-based strategy at *δ*_v_ = 0.98, leading to a probability of eradication of 0.01.

**Figure 5:**
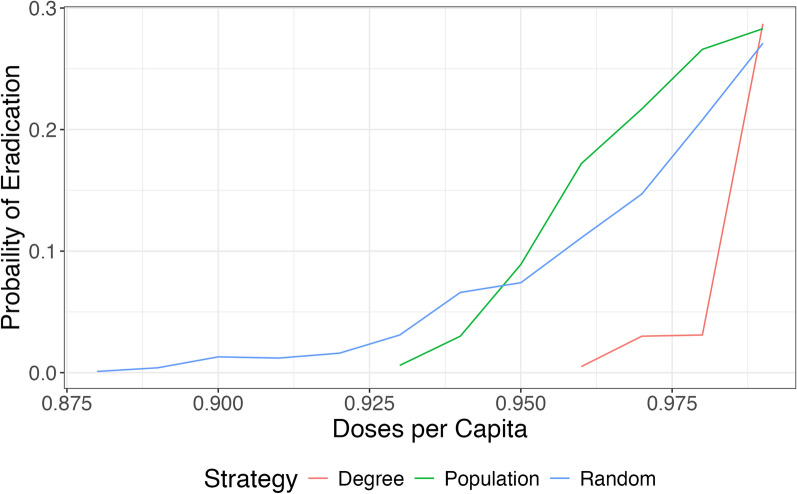
**Simulated probability of eradication for all strategies where eradication occurred, with vaccine efficacy at 90%.** Eradication is first seen with the random strategy at *δ*_v_ = 0.88 and increases to 0.29 as *δ*_v_ approaches 1. Population- and degree-based strategies also exhibit eradication at high dose availability (and efficacy); however, density-based allocation did not exhibit eradication at all.

With VE at 90%, three strategies resulted in eradication at high dose availability ratios (Figure 5) – Random, Population and Degree. Random sometimes led to eradication at the lowest dose availability (0.88), and all strategies peaked at a probability of eradication of approximately 0.29. Between *δ*_v_ = 0.95 and 1, population-based allocation led to a significantly higher probability of eradication than random allocation. Density-based allocation did not lead to eradication in any simulation.

**Figure 6:**
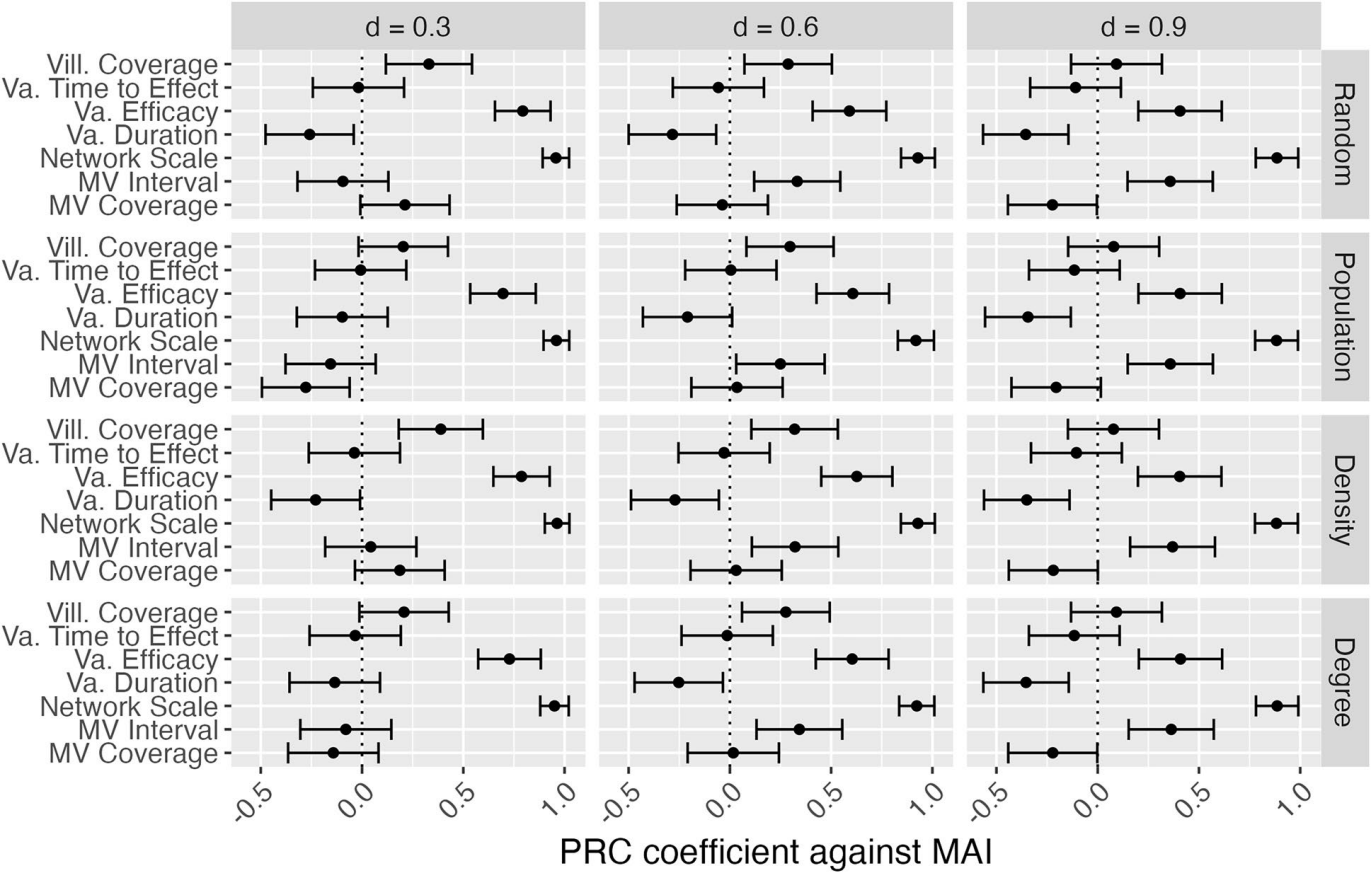
**PRC coefficients against mean annual incidence.** Each row is a different strategy (right), each column a different dose availability ratio.

Figure 6 shows the PRC coefficients of the sensitivity analysis against mean annual incidence. Results were sensitive to within-village coverage at low vaccine availability, but coefficients surrounded 0 at high availability. Network scale, representing the volume of cattle moving through the network, was significantly positively correlated with mean annual incidence for all vaccine availabilities and allocation strategies. For vaccine time to effect and vaccine duration, no coefficient was significantly different from 0 for any strategy or dose availability ratio. Vaccine efficacy was positively associated with incidence in every case, although the coefficient declined as dose availability increased. Coefficients for the mass vaccination interval were not significantly different from 0 at low dose availability, but positively associated with incidence at higher dose availability, although there appeared no difference by strategy. Mass vaccination coverage coefficients differed by dose availability and strategy: at low dose availability, it was negatively associated with incidence for the population- and degree-based strategies, but not significantly different from 0 for density-based allocation and slightly positively associated for random allocation; at medium dose availability no coefficients were significantly different from 0; at high dose availability, all strategies exhibited a strong negative association with incidence.

**Figure 7:**
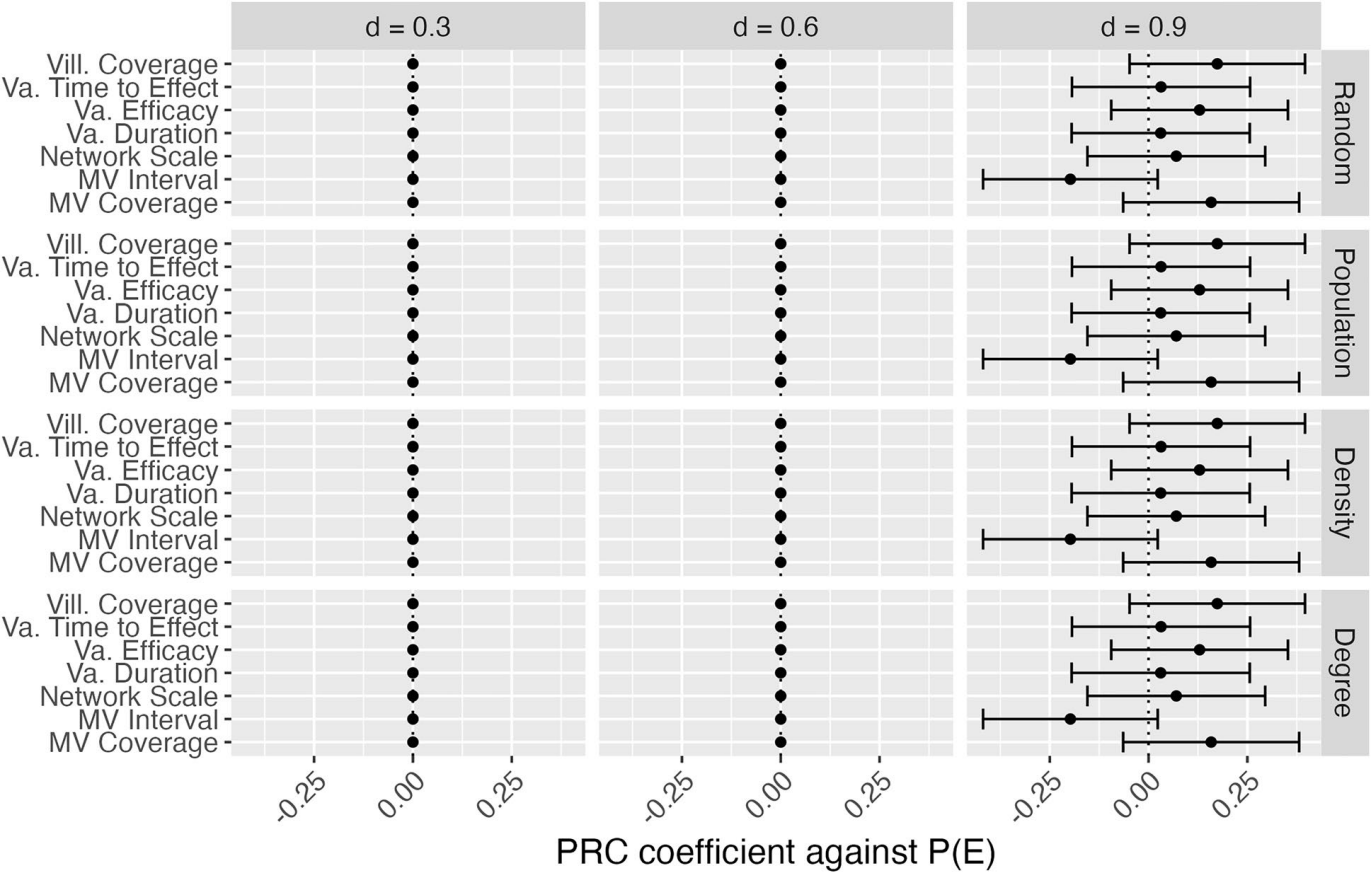
**PRC coefficients against probability of eradication.** Each row is a different strategy (right), each column a different dose availability ratio. No eradication was seen at *d* = 0.3 or 0.6, so coefficients are 0.

For probability of eradication, no eradication was seen at dose availability ratios below 0.88, so all coefficients for the low and medium availability scenarios were all 0 (Figure 7). For the high availability scenario and for all strategies, vaccine efficacy and mass vaccination coverage were positively associated with eradication, mass vaccination interval negatively associated and within village coverage, network scale, vaccine time to effect and duration not significantly different from 0.

## Discussion

In summary, the ‘optimal’ allocation strategy depends on the outcome being optimised for, the availability of vaccine doses and the efficacy of the vaccine being used. Random allocation at the herd level outperforms all other strategies at all dose availability ratios when optimising for average prevalence; however, if optimising for minimising incidence at low dose availability, a population-based strategy performs best. Eradication only occurs at very high dose availability with a highly effective vaccine, and the population-based allocation strategy maximises the probability of eradication. However, even with full coverage and a highly effective vaccine, accounting for parameter uncertainty the probability of eradication is still less than 0.3. When assessed against mean annual incidence, all strategies are sensitive to vaccine efficacy and mass vaccination coverage, and insensitive (within explored ranges) to vaccine time to protection and immunity duration.

The results indicate that random allocation of doses is the most effective strategy for most metrics investigated. It is strictly better than any other explored allocation strategy in reducing average disease prevalence, is somewhat better at reducing incidence at medium–high dose availability and was able to achieve eradication in some simulations at dose availability ratios lower than any other strategy. This surprising result does not appear to be due to the population structure of the simulated province (many smallholdings can lead to a high village coverage with a small animal coverage), as it also holds for prevalence at the level of the animal.

According to the results presented here, the second-best strategy is population-based dose allocation, which holds incidence lower than other strategies when few doses are available and maximises the probability of eradication at (very) high dose availability. Here, we must discuss the unintuitive pattern seen in Figure 4, where mean annual incidence increases before decreasing – this statistic is due to greater variability in observed incidence in the simulations. As more villages are vaccinated with greater dose availability, but not enough to truly eradicate the disease, when immunity wanes the village may be reinfected and recorded as a new infection.

Degree-based and density-based allocation strategies are less effective policies – they are neither best at reducing prevalence or incidence at any dose availability ratio. Degree-based (i.e. connectivity-based) allocation does, however, manage to achieve eradication when simulated with high dose availability and vaccine efficacy. The density-based strategy, somewhat surprisingly, never manages to achieve eradication even with enough doses for essentially the entire population. Close investigation revealed that, as there is little correlation between density and population in this landscape, there exist a set of medium-sized villages with approximately 6000 cattle who would never be vaccinated using this strategy except at 100% coverage – they were too far from every other village and hence were deprioritised in our simulations. This result may not generalise to other landscapes, where density and population may be positively correlated enough to avoid this failure mode.

All modelling results should be translated cautiously, and decisions must consider all available evidence and the unique characteristics of each country or region. However, these results suggest that in regions where the disease is currently endemic, and that wish to focus on control or eradication of FMD, it is better to first focus on achieving higher coverage or sourcing higher-quality vaccines than to attempt to ‘optimise’ where vaccines are allocated. If resources are available to optimise, however, our results suggest that:

1. If few vaccine doses are available, and the priority is to minimise incidence, vaccines should be allocated to the largest villages first. If the aim is to minimise average prevalence, random allocation should be used.
2. At high numbers of doses, population-based allocation maximises the probability of eradication.
3. Density-based allocation does not appear to offer any benefit over alternatives.

One limitation of these results that must be borne in mind is that, due to time and resource constraints, we only consider allocation of doses in a mass vaccination scenario. Such campaigns are generally carried out with other reactive control measures, such as ring vaccination around identified villages or livestock movement restrictions. Previous work of ours has addressed this [13]; however, we have not addressed the optimal allocation of doses between mass vaccination campaigns and reactive ring vaccination – we believe this would be interesting avenue for future research.

There is also the question of generality. Although our data from Turkey are high quality, and our model has demonstrated good fit to those data, the applicability of the model and results to other settings is unclear. Likewise, we have not included explicit seasonality into our model, incorporating this mainly through the seasonally varying movement data, and this may influence the results. The sensitivity analysis indicates that the average incidence is strongly affected by the volume of cattle movements taking place on the network, but no effect on probability of eradication, suggesting that this an important area for future work. These limitations should be considered before any policy decisions might be made.

We have focused on simple allocation strategies that we believe are straightforward to implement; however, we have not considered all possible allocation strategies. One that we did not consider, which is sometimes used, was risk-based spatial zoning – i.e. vaccinating as many animals as possible in a defined geographic area identified as high risk. Another interesting allocation strategy would be based on the friendship paradox, allocating randomly then further allocating to those villages that have recently sent or received animals, an approach which has been shown to have efficacy for surveillance [17]. It would be interesting to compare these strategies with these results.

Our results suggest that attempting to optimize vaccine allocation is, in many cases, counterproductive and leads to greater incidence and prevalence than using a simple random allocation strategy. They also caution against using density-based or degree-based allocation. Whilst our model could be extended to consider additional vaccine targeting strategies, we believe that this work can be a useful reminder that the problem of FMD can mostly be solved with enough high-quality vaccines applied at regular intervals.

## Supplementary Information

**Additional file 1** **Supplementary details**.

## Data Availability

The datasets supporting the conclusions of this article are available in the Zenodo repository under the reserved DOI **10.5281/zenodo.18471687** and will be made publicly available upon publication.

## References

[1] Arzt J, Juleff N, Zhang Z, Rodriguez LL (2011) The pathogenesis of foot-and-mouth disease I: viral pathways in cattle. Transbound Emerg Dis 58:291–304. 10.1111/j.1865-1682.2011.01204.x 21366894 10.1111/j.1865-1682.2011.01204.x

[2] Grubman MJ, Baxt B (2004) Foot-and-mouth disease. Clin Microbiol Rev 17:465–493. 10.1128/CMR.17.2.465-493.2004 15084510 10.1128/CMR.17.2.465-493.2004 PMC387408

[3] Knight-Jones TJD, Rushton J (2013) The economic impacts of foot and mouth disease – What are they, how big are they and where do they occur? Prev Vet Med 112:161–173. 10.1016/J.PREVETMED.2013.07.013 23958457 10.1016/j.prevetmed.2013.07.013 PMC3989032

[4] Govindaraj G, Kumar BG, Krishnamohan A, Hegde R,Kumar N, Prabhakaran K, Wadhwan VM, Kakker N, Lokhande T, Sharma K, Kanani A, Limaye, Natchimuthu K, Ananth PN, Kumar AD, Khan TA, Misri J, Dash BB, Pattnaik B , Habibur R (2021) Foot and mouth disease (FMD) incidence in cattle and buffaloes and its associated farm-level economic costs in endemic India. Prev Vet Med 190:105318. 10.1016/J.PREVETMED.2021.105318 33740596 10.1016/j.prevetmed.2021.105318

[5] Rasmussen P, Shaw AP, Jemberu WT, Knight-Jones T, Conrady B, Apenteng OO, Cheng Y, Muñoz V, Rushton J, Torgerson PR (2024 )Economic losses due to foot-and-mouth disease (FMD) in Ethiopian cattle. Prev Vet Med 230:106276 10.1016/J.PREVETMED.2024.106276 38991426 10.1016/j.prevetmed.2024.106276

[6] Leforban Y (1999) Prevention measures against foot-and-mouth disease in Europe in recent years. Vaccine 17:1755–1759. 10.1016/S0264-410X(98)00445-9 10194835 10.1016/s0264-410x(98)00445-9

[7] Rivera AM, Sanchez-Vazquez MJ, Pituco EM, Buzanovsky LP, Martini M, Cosivi O (2023) Advances in the eradication of foot-and-mouth disease in South America: 2011–2020. Front Vet Sci 9:1024071. 10.3389/FVETS.2022.1024071 36699326 10.3389/fvets.2022.1024071 PMC9868265

[8] Hammond JM, Maulidi B, Henning N (2021) Targeted FMD vaccines for Eastern Africa: the AgResults foot and mouth disease vaccine challenge project. Viruses 13:1830. 10.3390/V13091830 34578411 10.3390/v13091830 PMC8472200

[9] Keeling MJ, Woolhouse MEJ, May RM, Davies G, Grenfell BT (2003) Modelling vaccination strategies against foot-and-mouth disease. Nature 421:136-142. 10.1038/nature01343 10.1038/nature01343 12508120

[10] Tildesley MJ, Savill NJ, Shaw DJ, Deardon R, Brooks SP, Woolhouse MEJ, Grenfell BT, Keeling MJ (2006) Optimal reactive vaccination strategies for a foot-and-mouth outbreak in the UK. Nature 440:83–86. 10.1038/nature04324 16511494 10.1038/nature04324

[11] Roche SE, Garner MG, Sanson RL, Cook C, Birch C, Backer JA, Dube C, Patyk KA, Stevenson MA, Yu ZD, Rawdon TG, Gauntlett F (2015) Evaluating vaccination strategies to control foot-and-mouth disease: a model comparison study. Epidemiol Infect 143:1256–1275. 10.1017/S0950268814001927 25078780 10.1017/S0950268814001927 PMC9507177

[12] Ringa N, Bauch CT (2014) Impacts of constrained culling and vaccination on control of foot and mouth disease in near-endemic settings: a pair approximation model. Epidemics 9:18–30. 10.1016/J.EPIDEM.2014.09.008 25480131 10.1016/j.epidem.2014.09.008

[13] Guyver-Fletcher G, Gorsich EE, Jewell C, Tildesley MJ (2025) Controlling endemic foot-and-mouth disease: vaccination is more important than movement bans. A simulation study in the Republic of Turkey. Infect Dis Model 10:702–715. 10.1016/J.IDM.2025.02.006 40091911 10.1016/j.idm.2025.02.006 PMC11907466

[14] Stenfeldt C, Arzt J (2020) The carrier conundrum; a review of recent advances and persistent gaps regarding the carrier state of foot-and-mouth disease virus. Pathogens 9:167. 10.3390/PATHOGENS9030167 10.3390/pathogens9030167 PMC7157498 32121072

[15] Knight-Jones TJD, Bulut AN, Gubbins S, Stark KDC, PfeifferDU, Sumption KJ, Paton DJ (2014) Retrospective evaluation of foot-and-mouthdisease vaccine effectiveness in Turkey. Vaccine 32:1848–1855. 10.1016/J.VACCINE.2014.01.071 24530150 10.1016/j.vaccine.2014.01.071 PMC3991324

[16] Stevenson M, Sergeant E (2025) epiR: Tools for the analysis of epidemiological data. 10.32614/CRAN.package.epiR

[17] Amaku M, Grisi-Filho JHdeH, Negreiros RL, Dias RA, FerreiraF, Neto JSF, Cipulla RI, Marques FS, Ossada R (2015) Infectious disease surveillance inanimal movement networks: an approach based on the friendship paradox. Prev VetMed 121:306–313.10.1016/J.PREVETMED.2015.08.002 26277202 10.1016/j.prevetmed.2015.08.002

