# Supplementary material for: Assessing the effects of limited vaccine supply on risk-based vaccination strategies in regions of endemic foot-and-mouth disease
Source: Vet Res. 2026 Jul 7;57:126. doi: 10.1186/s13567-026-01805-y (PMC13340340; doi:10.1186/s13567-026-01805-y)
Supplement: Supplementary file 1 — Additional file 1 Supplementary details. [file 13567_2026_1805_MOESM1_ESM.docx]

# Supplementary details for “Assessing the effects of limited vaccine supply on risk-based vaccination strategies in regions of endemic foot-and-mouth disease.”

## Description of model

We utilise a stochastic spatial metapopulation model, where each village is considered a separate population and the within-village and between-village dynamics are modelled interdependently. The model structure and baseline parameterisation follow that described in Guyver-Fletcher et al, 2025; we summarise here for completeness.

### Within-village

Within each village, cattle are divided into several mutually exclusive epidemiological classes: maternally immune (M); susceptible (S); exposed (E); infectious (I); recovered (R); carrier (C); vaccinated-recovered (VR); vaccinated-susceptible (VS). Cattle transition between these disease states, governed by ODEs implemented stochastically using the tau-leap algorithm to approximate a continuous-time Markov process. Births introduce animals into the maternally immune class or the susceptible class depending on the proportion of the population that have immunity. Infection occurs through frequency-dependent transmission within villages, with exposed animals progressing to the E and then I classes. Carrier animals are assumed not to be infectious, consistent with current evidence.

At the beginning of the model, all animals within all villages are assumed susceptible, progressing between states *q* and *r* according to rate $\lambda_{i}^{qr}(t)$ so that:


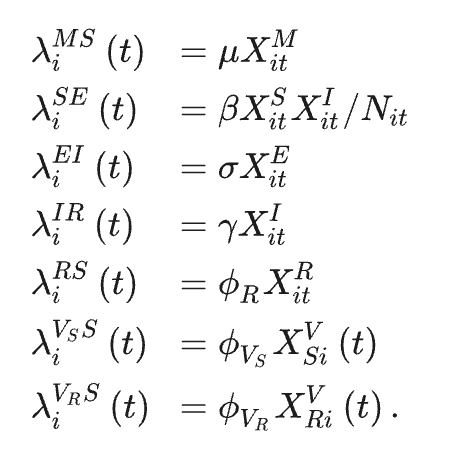


The parameters of which are described in the table below.

| Parameter | Description | Value(s) | Source(s) |
| --- | --- | --- | --- |
| $\boldsymbol{\alpha}$ | Birth rate | 2%/year |  |
| $\boldsymbol{\beta}$ | Transmission | 6/11 transmissions per contact | Orsel et al, 2005; Orsel et al, 2007; Tadesse et al, 2019 |
| $\boldsymbol{\gamma}^{\boldsymbol{-}\boldsymbol{1}}$ | Mean infectious period | 11 days | Yadav et al, 2019 |
| $\boldsymbol{\phi}_{\boldsymbol{R}}^{\boldsymbol{-}\boldsymbol{1}}$ | Mean time recovered | 250 days | El-Sayed et al, 2012; Doel 2005 |
| $\boldsymbol{\mu}^{\boldsymbol{-}\boldsymbol{1}}$ | Mean duration of maternal immunity | 120 days | Nicholls et al, 1984 |
| $\boldsymbol{\sigma}^{\boldsymbol{-}\boldsymbol{1}}$ | Mean latent period | 1.5 days | Yadav et al, 2019 |
| $\boldsymbol{\Omega}_{\boldsymbol{d}}$ | Infection mortality rate | 2% | Senturk & Yalcin, 2008 |
| $\boldsymbol{\Omega}_{\boldsymbol{n}}$ | Natural mortality rate | 2% rate |  |
| $\boldsymbol{\phi}_{\boldsymbol{V}_{\boldsymbol{S}}}^{\boldsymbol{-}\boldsymbol{1}}$ | Mean time vaccine-immune | 150 days | Doel 2003; Cox et al 2010; Knight-Jones et al, 2015 |
| $\boldsymbol{\phi}_{\boldsymbol{V}_{\boldsymbol{R}}}^{\boldsymbol{-1}}$ | Mean time vaccine-immune (Recovered) | Max**(** $\phi_{R}^{-1}$, $\phi_{V_{R}}^{-1}$**)** | Doel 2003; Cox et al 2010; Knight-Jones et al, 2015 |
| $\boldsymbol{\rho}$ | Vaccine delay days | 4 days | Barnett & Carabin, 2002 |

Vaccination moves a proportion of susceptible and recovered animals into their respective vaccinated compartments, with protection occurring after a delay and waning back into the susceptible state.

### Between-village transmission

There are two distinct mechanisms through which inter-village transmission is modelled. First, direct cattle movements are simulated explicitly using the available movement data. The probability of seeding infection in a sink village is proportional to the number of animals moved and the prevalence of infection in the source village.

Second, a distance-dependent kernel (equation below) captures the indirect and short-range transmission routes, including aerosol spread and unobserved contacts. The force of infection between two villages declines with increasing geographic separation and increases with the number of infectious animals in the source village, as well as the number of susceptible animals in the sink village.

$$p_{i}\left( t \right)=1- e^{-\zeta\sum_{j\neq i} X_{jt}^{I}K\left( d_{ij} \right)}$$

$$K\left( d_{ij} \right)= \frac{1}{1+\left( \frac{d_{ij}}{\nu} \right)^{\eta}}$$

$d_{ij}$ is the Euclidean distance between village centroids, $\zeta$ is a baseline inter-village transmission rate, and $\nu$ and $\eta$ govern how local spread decays with distance. Values used are sourced from the posterior distribution of Guyver-Fletcher et al, 2025.

### Mass Vaccination

Simulations include the option for control policies when specified. Mass vaccination is modelled as occurring on fixed intervals (e.g. every 182 days), at which villages are targeted for vaccination and ordered according to the desired criteria (random – i.e. shuffled, population, density, degree). The number of ‘available’ vaccine doses at that moment is calculated (doses per capita multiplied by the number of animals at time t). The village at the top of the ordered list is vaccinated and the number of doses used is subtracted from the doses in stock, until either all villages are vaccinated or there is insufficient vaccine doses left to vaccinate the village next on the list. When a village is vaccinated, the ‘true’ efficacy of the vaccine (i.e. the proportion of the vaccinated population that is assumed to result in protection) is drawn from a normal distribution centred on the average efficacy specified for the vaccine.

## Marginal Prevalence Elasticity

**Figure 1** demonstrates the marginal prevalence elasticity for each strategy – a measure of how responsive prevalence is to a change in vaccine availability. At both vaccine efficacies, the random strategy exhibits a fairly constant elasticity, slowly improving from approximately –0.8 to –1.0 between δ_v_ = 0 to 0.5, and improving faster to approximately –2 (VE = 71) or -3.7 (VE=90) as δ_v_ approaches 1.0. In contrast, the population-based allocation strategy starts at approximately 0, exhibiting no effect on simulated prevalence, until δ_v_ exceeds 0.25, before more rapidly improving to approximately –3.5 at δ_v_ = 0.9 and then climbing to –2 at δ_v_ = 1.0.


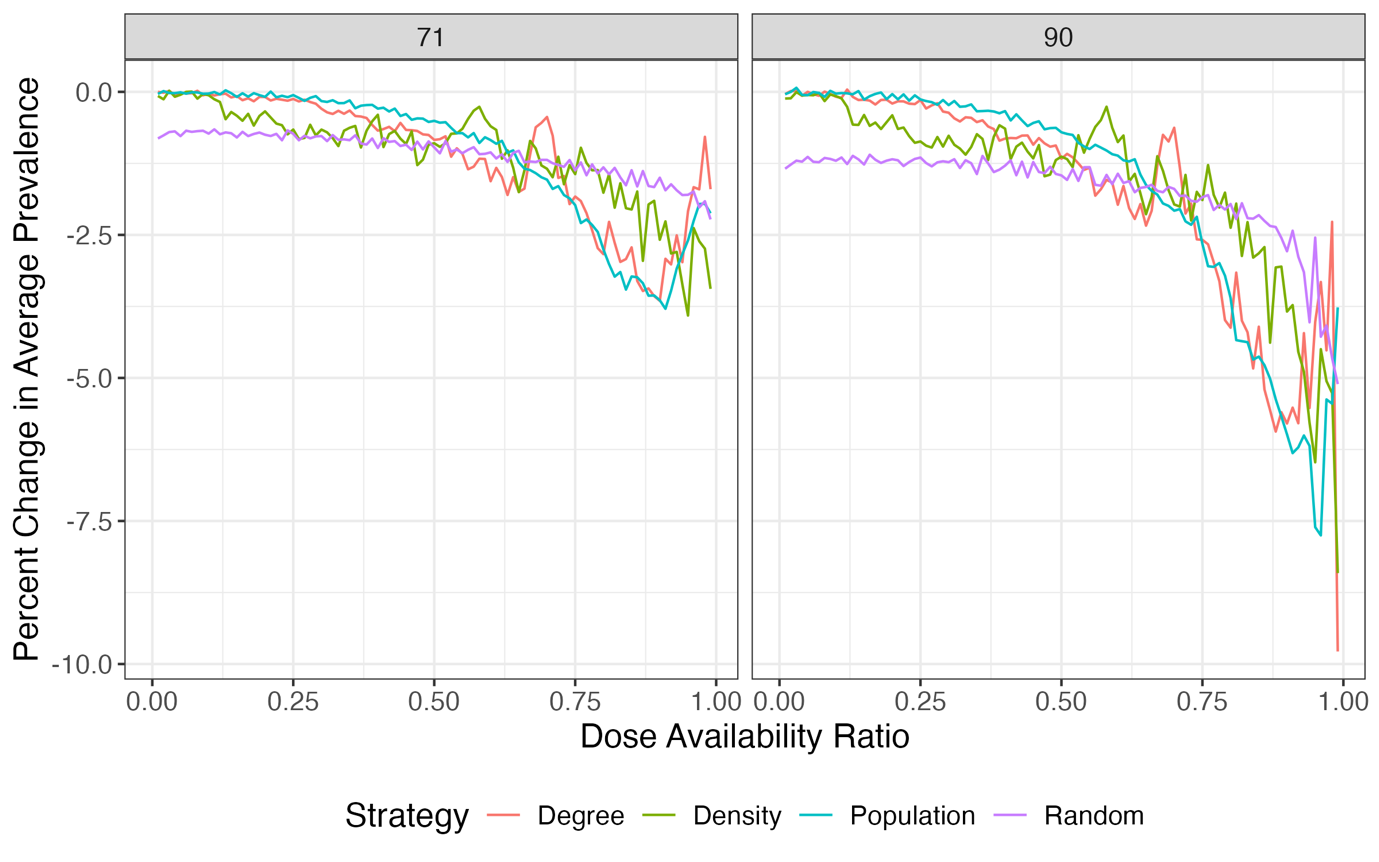


**Figure 1: Prevalence Elasticity by dose availability ratio and allocation strategy.** The percentage effect on prevalence of a 1 percentage point increase in dose availability ratio at VE = 71 or 90 (facets). Lower numbers are better. For example, a value of -2 indicates that a 1% increase in dose availability leads to a 2% decrease in predicted prevalence. All strategies exhibit declines of less than 1% prevalence at δ_v_ < 0.6, with greater elasticity at greater dose availability. Much greater elasticity is observed in the greater vaccine efficacy scenario.

Non-random allocation strategies exhibit lesser prevalence elasticity at low dose availabilities, but greater elasticity at higher dose availabilities. This is likely due to the target nature of the allocation strategies – at low doses there are few villages being vaccinated, so prevalence cannot move much, but at high dose availability they are `enrolling` a greater proportion of the villages with each increase and can consequently have a much greater response in terms of reduction in prevalence.

## Sensitivity of Mean Time to Eradication

For mean time to eradication, no coefficients were significantly different from 0, even at 90% dose availability (**Figure 2**).


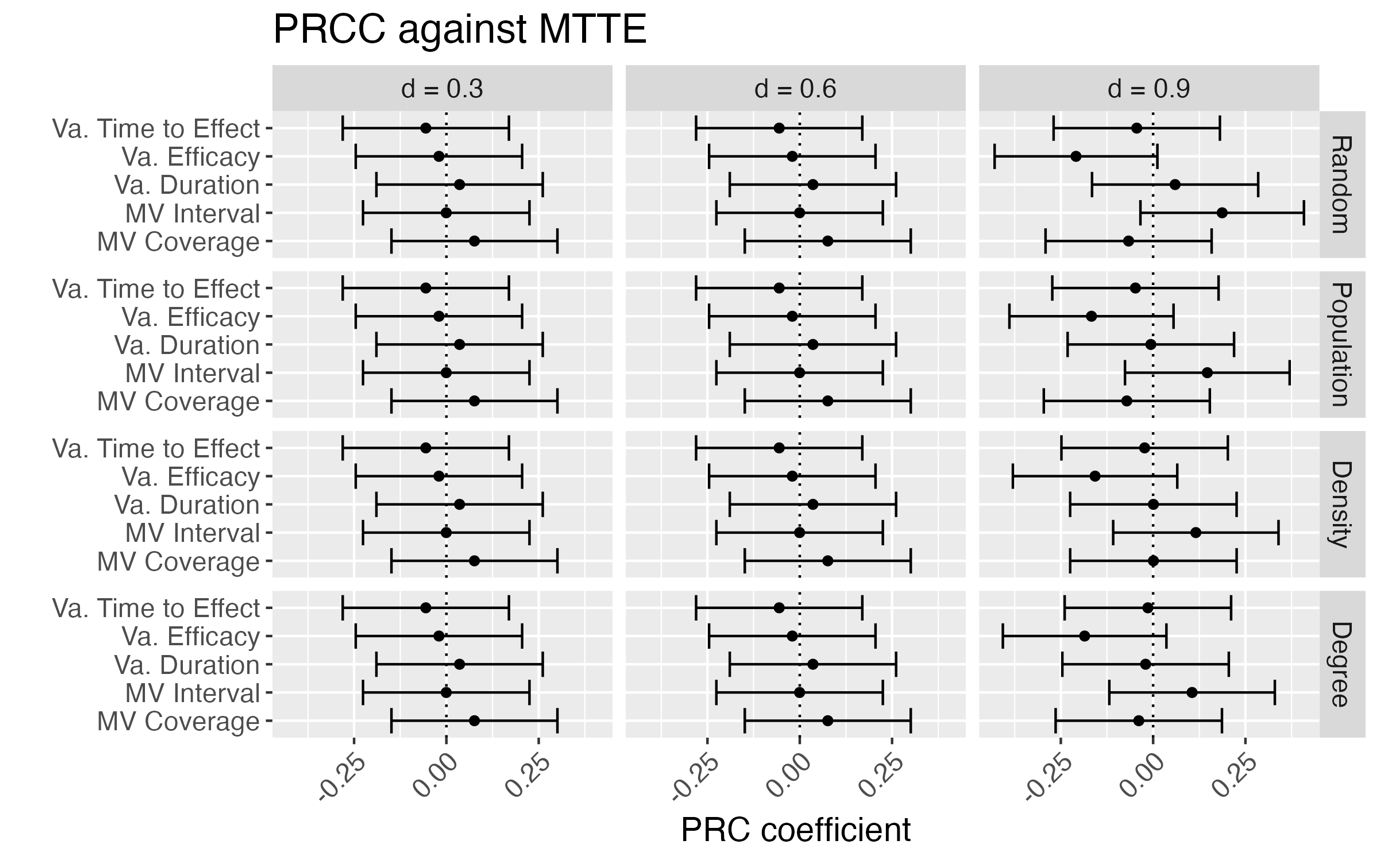


**Figure 2: PRC coefficients for Mean Time to Eradication.** Each row is a different strategy (right), each column a different dose availability ratio.
